# Mechanism by which porcine transmissible gastroenteritis virus disrupts host innate immunity

**DOI:** 10.3389/fimmu.2025.1675572

**Published:** 2025-09-25

**Authors:** Zian Wang, Jiahao Xie, Qindan Li, Yiheng Liu, Xiaotao Zhang, Ergen Mi, Liang Wang, Lingyu Wang, Feng Zhang

**Affiliations:** ^1^ Department of Gastrointestinal Surgery, The Second Hospital of Dalian Medical University, Dalian, Liaoning, China; ^2^ Advanced Institute for Medical Science, Dalian Medical University, Dalian, Liaoning, China; ^3^ Research and Teaching Department of Comparative Medicine, Dalian Medical University, Dalian, Liaoning, China

**Keywords:** TGEV, immune response, innate immune escape, PRRs, PAMPS, IFN

## Abstract

Innate immune evasion is a critical aspect of viral infections, as it disrupts the host’s defense mechanisms.The innate immune system, as the primary defense against pathogens, detects pathogen-associated molecular patterns (PAMPs) via pattern recognition receptors (PRRs). This recognition triggers the production of interferons (IFNs) and pro-inflammatory factors, initiating the antiviral immune response. During evolution, viruses have found many ways to evade innate immune response in order to increase the replication efficiency, transmission ability and to establish persistent infection through co-evolution with hosts. Pigs act as natural hosts for a variety of significant viruses, including both DNA and RNA viruses. These viruses not only jeopardize animal health but also present a potential risk of interspecies transmission. Among these, porcine transmissible gastroenteritis virus (TGEV) stands out as a highly prevalent and severely detrimental enterovirus in the global swine industry. This review aims to comprehensively analyze the interaction between TGEV and host cells, emphasizing the molecular underpinnings of its immune evasion strategies. In addition, we will describe the programmed cell death types induced by TGEV, including autophagy, apoptosis and pyroptosis. Compared with existing reviews, this article not only provides a systematic integration of the multilayered immune evasion mechanisms of TGEV but also, for the first time, offers a comprehensive overview of its interactions with various forms of programmed cell death. This perspective highlights the complex regulatory networks underlying TGEV’s adaptive evolution in the host, thereby enhancing our understanding of the pathogenic mechanisms of porcine coronaviruses and offering novel theoretical foundations for the development of vaccines and antiviral therapeutics.

## 1 Introduction

**Figure 1:**
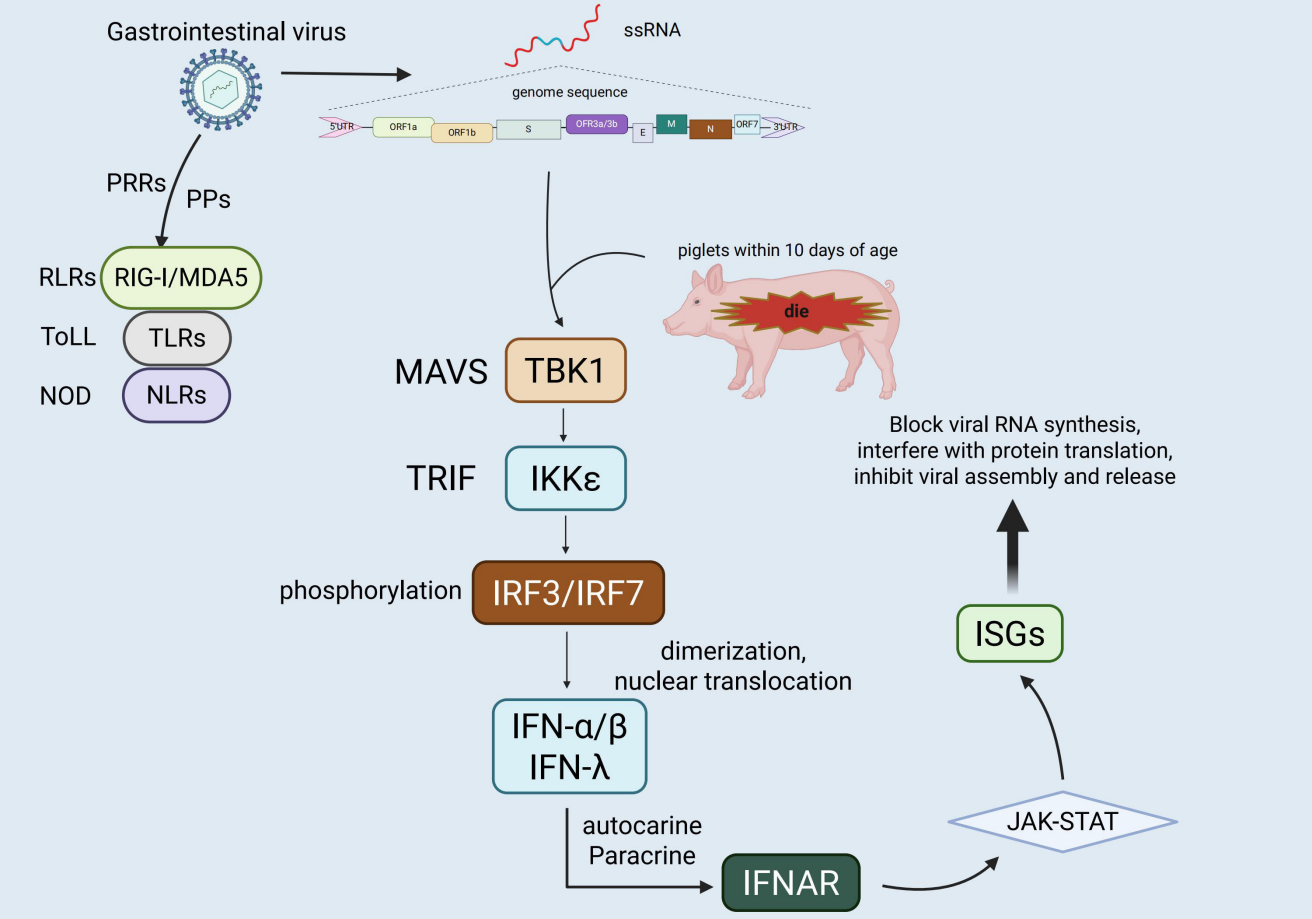
Genomic organization of TGEV and host innate immune responses. TGEV is an enveloped, positive-sense single-stranded RNA virus whose genome is organized as 5′UTR–ORF1a–ORF1b–S–ORF3a/3b–E–M–N–ORF7–3′UTR. It encodes four structural proteins,*namely* the spike (S), envelope (E), membrane (M), and nucleocapsid (N)—as well as several accessory proteins. In piglets *less than* 10 days of age, infection typically causes severe diarrhea and is often fatal, with mortality rates reaching 80–100%. The figure also illustrates the recognition of viral RNA by host pattern recognition receptors (TLRs, RLRs, and NLRs), *along with* the activation of downstream interferon signaling pathways. These pathways induce antiviral gene expression and contribute to host defense.

As the first line of defense against the invasion of exogenous pathogens, the innate immune system is characterized by rapid recognition and broad responses (1, 2).Central to this system is the detection of PAMPs by pattern recognition receptors (PRRs),which initiates an immune response against infections (3, 4). In the context of viral infections, typical PAMPs of viruses include their nucleic acids, such as single - stranded RNA(ssRNA,including 5’ UTR, viral RNA and replication protein), double - stranded RNA (dsRNA), and DNAThese PAMPs are recognized by different PRRs of host cells (5, 6) (including, but not limited to, TLRs, RIG-I-like receptors (RLRs), the cGAS-STING pathway and NOD-like receptors (NLRs)) (7–9). Upon recognizing viral components, these receptors activate downstream transcription factors IRF3/7 and NF-κB through adaptor proteins (e.g., MAVS, TRIF, MyD88, STING), This activation process ultimately leads to the production of type I interferons (IFN-α/β) and pro-inflammatory cytokines. These molecules can effectively inhibit viral replication and trigger adaptive immune responses (10–12). However, over time, viruses have evolved diverse mechanisms to evade the innate immune system, collectively referred to as “innate immune escape” (13, 14). These strategies work by interfering with the recognition of viral nucleic acids by pattern recognition receptors, preventing the activation of adaptor proteins or key signaling pathways, and promoting the expression of host negative regulatory factors to suppress the immune response (15, 16). These evasion tactics are crucial for successful viral infection, immune evasion, and also provide the molecular basis for interspecies transmission and viral pandemics (**Figure 1**).

Among diverse viruses, porcine coronaviruses—particularly TGEV—serve as a representative model for investigating viral immune evasion mechanisms. On one hand, TGEV shares similarities in genomic organization and immune regulatory strategies with other coronaviruses, such as SARS-CoV-2, thereby providing valuable insights into the conserved mechanisms underlying coronavirus immune escape (17). On the other hand, as a highly pathogenic enteric virus that poses a serious threat to the swine industry, TGEV not only impairs animal health and livestock production but also harbors potential risks of cross-species transmission (18).

To date, six porcine coronaviruses have been identified, comprising four alphacoronaviruses, one betacoronavirus, and one deltacoronavirus (19). Among them, TGEV is the earliest discovered and most extensively investigated member of the porcine alphacoronaviruses (20). Viral structural proteins not only mediate essential functions in the viral life cycle, including entry, assembly, and budding, but also play pivotal roles in orchestrating immune evasion strategies, forming the molecular basis by which TGEV circumvents host innate immune recognition (21).TGEV is transmitted primarily via the fecal–oral and respiratory routes, infecting epithelial cells of the porcine gastrointestinal tract, particularly the villous epithelial cells of the small intestine (21, 22). Viral replication results in epithelial cell necrosis, villus atrophy, and mucosal damage, leading to disruption of the intestinal barrier. Consequently, infected piglets exhibit acute vomiting, profuse yellow-green watery diarrhea, severe dehydration, and malabsorption (23, 24). The disease is especially devastating in neonatal piglets with immature immune systems, in which rapid disease progression is associated with mortality rates as high as 80–100% within the first 10 days of life (25). Infected pigs, asymptomatic carriers, and animals within 10 weeks of recovery serve as major sources of transmission. To date, pigs are recognized as the only natural host of TGEV, with no human infections reported.

The high pathogenicity and rapid transmission of TGEV pose a substantial threat to the swine industry. Elucidating the molecular mechanisms underlying TGEV immune evasion not only offers critical insights into the general pathogenic strategies of coronaviruses but also guides vaccine development and antiviral drug design. Compared with other porcine alphacoronaviruses, such as Porcine Epidemic Diarrhea Virus (PEDV), which also causes severe diarrhea, and the recently identified SADS-CoV, which exhibits cross-species transmission potential, TGEV displays distinctive features in terms of pathogenicity, tissue tropism, and immune evasion strategies. These differences offer unique opportunities for comparing the mechanisms of pathogenesis among porcine coronaviruses, highlighting the importance of systematic investigation of TGEV immune evasion in understanding coronavirus evolution, assessing interspecies transmission risks, and advancing novel prevention and control strategies.

## 2 Recognition and response of the host’s innate immunity

The innate immune system of pigs, like that of other mammals, relies on the recognition of PAMPs by PRRs for its functionality (26, 27). Upon invasion by enteroviruses such as TGEV, various PRRs expressed in porcine intestinal epithelial cells and mucosa-associated immune cells play a crucial role in detecting the nucleic acid components of different viruses, thereby initiating an antiviral innate immune response. Among them, the RLRs located in the cytoplasm are key sensors for RNA virus recognition, including Retinoic Acid-Inducible Gene I(RIG-I) and Melanoma Differentiation-Associated Gene 5(MDA5), which recognize 5’-triphosphate single - stranded RNA and long - chain double - stranded RNA respectively (28, 29). TLRs located on the endosomal membrane are also significant in virus recognition. For instance, TLR3 detects double-stranded RNA generated during viral replication, while TLR7/8 primarily identify single-stranded RNA, both contributing to impeding virus replication and dissemination (30, 31). Moreover, although the cGAS-STING pathway is primarily involved in detecting cytoplasmic DNA, predominantly against DNA viruses, it can be indirectly activated during RNA virus infections by cellular damage or secondary signals, thereby participating in innate immune modulation (32, 33). Furthermore, in addition to their role in inflammasome assembly and pro-inflammatory factor release, NLRs can act as co-regulators of PRRs signaling pathways, augmenting immune recognition and response to viral infections (34, 35).

**Figure 2:**
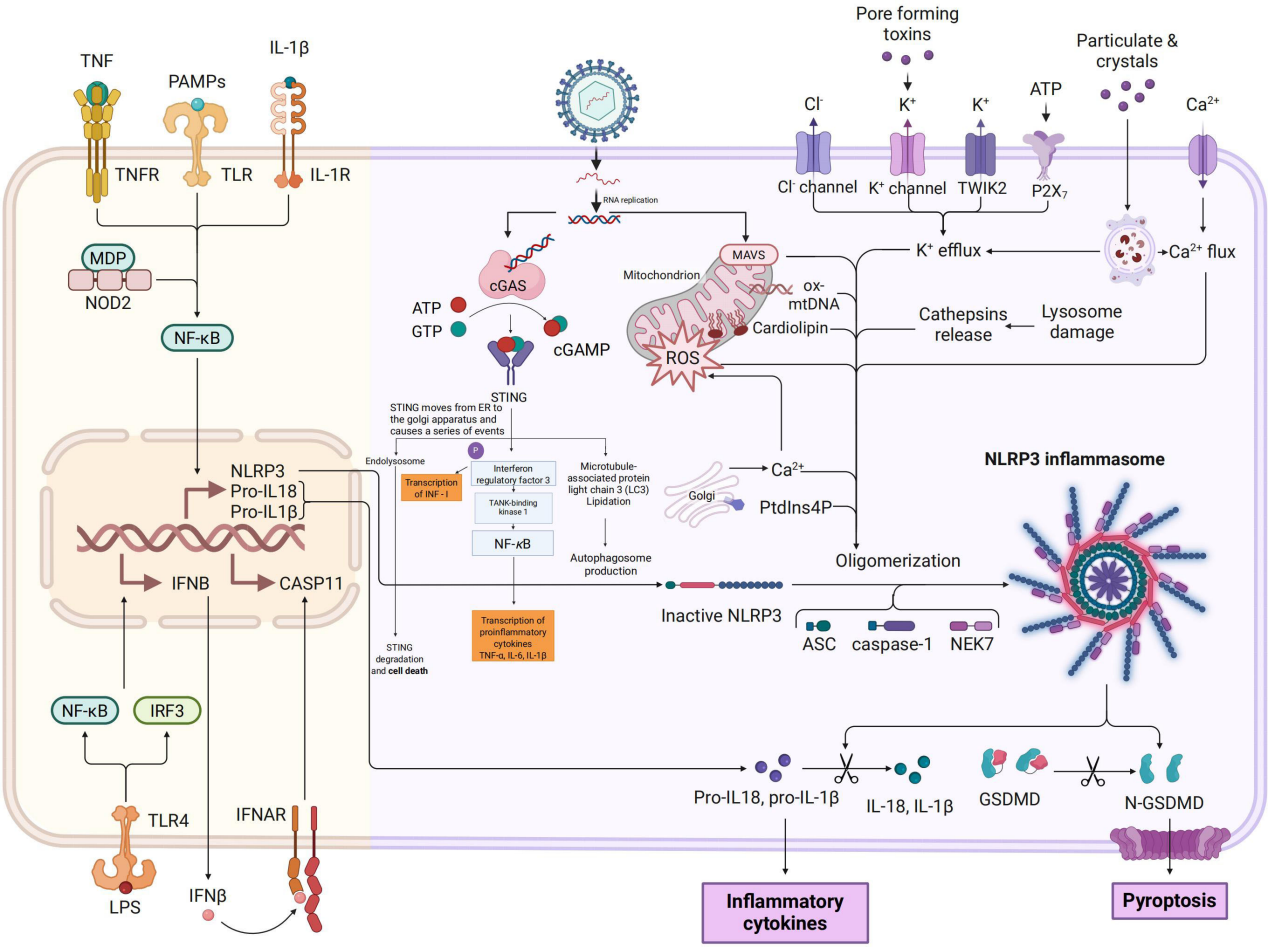
Schematic representation of NLRP3 inflammasome and cGAS-STING pathway activation. This figure *illustrates* the activation mechanisms and downstream effects of the NLRP3 inflammasome (on the left) and the cGAS-STING pathway (on the right), highlighting their interconnections in innate immune responses. The left panel shows that priming signals, which are mediated by TNFR, TLR, and IL-1R upon stimulation with TNF, PAMPs, and IL-1β, lead to NF-κB activation and NLRP3 inflammasome assembly. This assembly process involves nucleotide - binding oligomerization domain - containing protein 2 (NOD2), muramyl dipeptide (MDP), ATP, K^+^ efflux, and ROS, ultimately inducing caspase - 11 (CASP11) and interferon - β (IFNB) expression, pyroptosis, and IL-1β release. The right panel illustrates cyclic GMP - AMP synthase (cGAS) binding to cytosolic DNA to generate cGAMP, which activates STING translocation from the endoplasmic reticulum (ER)to the Golgi and subsequently triggers the activation of interferon regulatory factor 3 (IRF3) and NF-κB, resulting in antiviral responses and pro-inflammatory cytokine production. Shared regulatory elements include ROS and Ca²^+^ perturbations, mitochondrial damage, and lysosomal rupture, with organelles such as lysosomes, mitochondria, and autophagosomes contributing to inflammasome activation, pyroptosis, and cytokine synthesis.

Upon viral infection, host cells detect viral nucleic acids via PRRs, leading to the recruitment of specific adapter proteins (e.g., MAVS, TRIF, MyD88). These adapter proteins subsequently activate the downstream signaling molecules TANK-binding kinase 1 (TBK1) and IKKϵ, which phosphorylate transcription factors IRF3 and IRF7 (36–38). This phosphorylation prompts the dimerization and nuclear translocation of IRF3 and IRF7, thereby initiating the transcriptional expression of type I interferons (IFN-α/β) and type III interferons (IFN-λ) (39, 40). In the context of antiviral immunity in the intestinal mucosa, type III interferons exhibit greater tissue specificity and targeting compared to type I interferons (41). Due to the predominant expression of its receptor (IFNLR1/IL10R2) on epithelial cells, IFN-λ enhances local antiviral defense effectively while limiting inflammation, thereby playing a pivotal role in maintaining intestinal immune homeostasis and controlling local viral infections (42, 43). Subsequently, the secreted interferons bind to their respective receptors on target cells through autocrine and paracrine mechanisms (type I interferons bind to IFNAR1/2, and type III interferons bind to IFNLR1/IL10R2), activating the JAK-STAT signaling pathway and inducing the expression of numerous interferon-stimulated genes (ISGs) (44–46). These ISGs can impede various stages of the viral life cycle, including viral RNA synthesis, protein translation, and viral assembly and release, collectively establishing an effective innate antiviral barrier (47, 48). However, neonatal piglets under one week of age, characterized by an immature immune system, exhibit low expression levels of PRRs and downstream signaling molecules, limiting the efficiency of the interferon response. Consequently, this inadequate response fails to promptly control viral infections in the early stages, contributing significantly to their heightened susceptibility to enteric viruses like TGEV and the associated elevated mortality rate (49), **Figure 2**.

## 3 Interference of TGEV with host pattern recognition receptors

### 3.1 Mechanistic insights into TGEV NSP-mediated blockade of TLR/RLR signaling

TLRs and RLRs are crucial PRRs in the innate immune system, responsible for detecting viral nucleic acids (50, 51). The single-stranded positive-sense RNA genome of TGEV is recognized by TLR3 and TLR7/8 localized in endosomes or by RIG-I and MDA5 localized in the cytoplasm to activate the downstream signaling pathways leading to the expression of type I IFNs (IFN-α/β) and pro-inflammatory mediators (52, 53). The transcription of these two types of IFNs is mediated by the activation of either IRF3/7 or NF-κB, which are pivotal for initiating antiviral immune responses (54). To evade host immune surveillance effectively, TGEV has developed diverse immune evasion tactics, predominantly utilizing its NSPs to disrupt key components of the TLR and RLR signaling pathways. This interference hampers interferon production and innate immune activation (55, 56).

**Figure 3:**
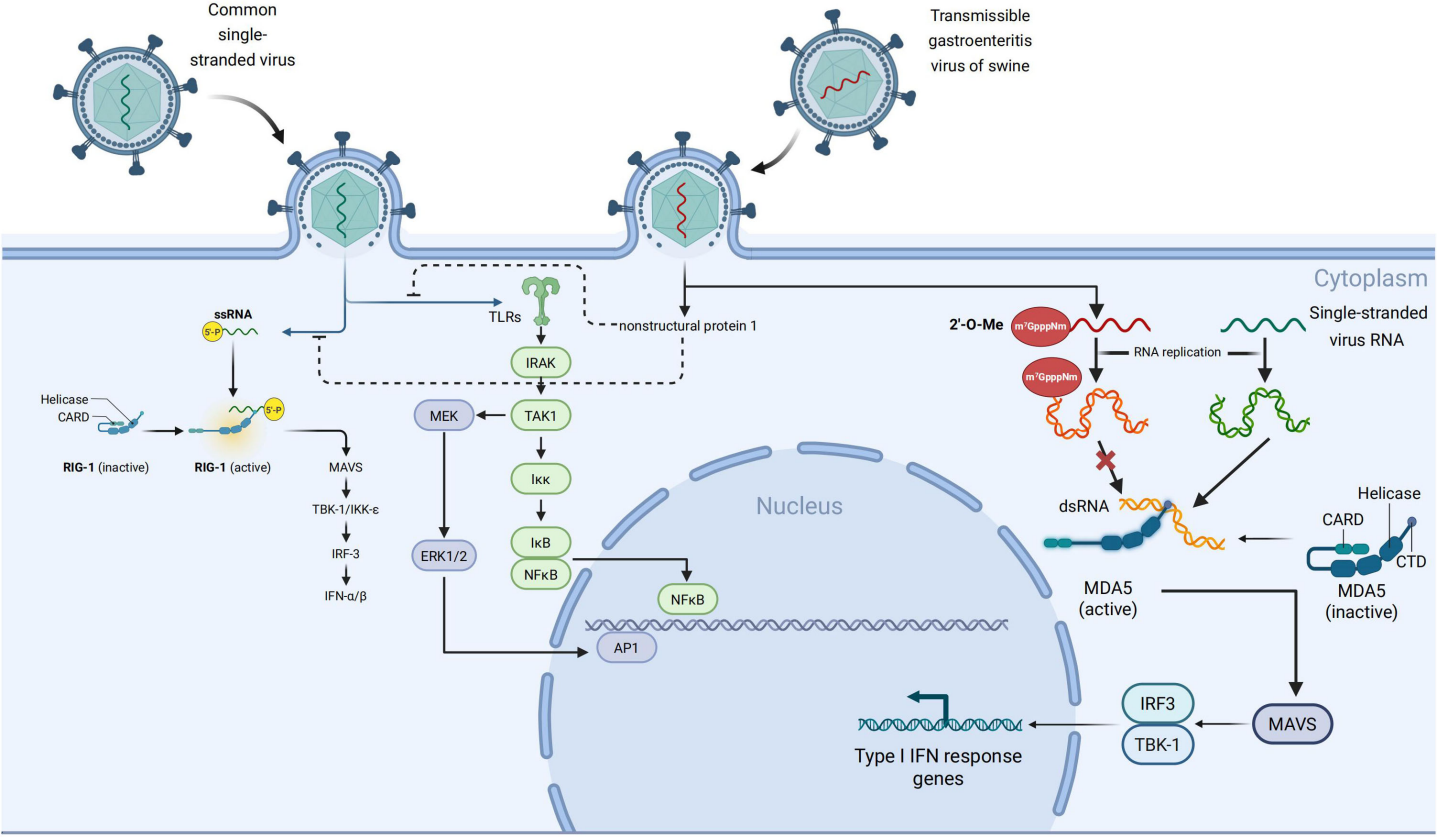
Mechanisms by which TGEV evades host PRRs-mediated innate immune responses. In uninfected cells, viral RNA is recognized by endosomal Toll-like receptors (TLR3/TLR7) or cytoplasmic sensors such as Melanoma Differentiation - Associated Gene 5 (MDA5). This recognition triggers downstream signaling cascades via adaptor kinases and transcription factors, ultimately leading to the production of type I interferons and pro - inflammatory cytokines.However, during TGEV infection, viral non-structural proteins (NS) inhibit key signaling nodes by promoting protein degradation or functional inactivation, thereby disrupting the TLR/RLR signaling pathways. This results in impaired nuclear translocation of IRF3 and NF-κB,along with reduced cytokine expression. In addition, TGEV evades MDA5 recognition by modifying the viral RNA cap structure through 2’-O methylation (m^7^GpppNm), which prevents MDA5 binding and subsequent activation of downstream signaling.

During the initial phase of TGEV infection, the viral genomic open reading frame ORF1a is translated into polyprotein pp1a, which is further extended and translated into pp1ab through -1 ribosomal frameshifting (57, 58). These polyproteins are later processed by viral proteases, namely papain-like protease (PL^pro^) and 3C-like protease (3CL^pro^), resulting in the generation of 16 non-structural proteins such as NSP1 and NSP3 (59–61). These proteins are pivotal in virus replication, host modulation, and immune evasion. NSP1 is one of the earliest expressed viral proteins and has obvious immunosuppressive functions (62). This protein can inhibit the translation process by blocking the binding of host mRNA to ribosomes (63) and may promote the degradation of host mRNA through an as-yet-unclear mechanism (64, 65). NSP1 has been shown to hinder the functional activation of IRF3 by promoting its degradation. Normally, IRF3 is phosphorylated upon viral infection, leading to its dimerization, nuclear translocation, and subsequent induction of IFN and ISG expression (66, 67). TGEV’s NSP1 disrupts the IFN-β signaling pathway by targeting various steps of the IRF3 pathway, thereby obstructing IRF3 activation, nuclear translocation, and binding to target gene promoters, ultimately suppressing type I interferon production to facilitate immune evasion (68, 69). Moreover, NSP1 interferes with host mRNA transcription and translation processes, exacerbating the inhibition of host antiviral protein synthesis and enhancing immune evasion (70). It also promotes virus replication by impeding stress granule (SG) formation (71). NSP3 of TGEV is a multifunctional protein. In addition to participating in the self-cleavage of viral polyproteins, it also has a PLpro domain and deubiquitinase (DUB) activity, and can target multiple host immune signaling molecules for regulation (72, 73). PLP domain can directly recognize and cleave TRAF3. TRAF3 is an important adaptor protein located downstream of the MAVS in RLR signaling pathway and is responsible for recruiting and activating TBK1 and IKKϵ kinase complex (74). Once TRAF3 is cleaved, the formation of the TBK1-IKKϵ complex is impaired, leading to reduced phosphorylation of IRF3 and IRF7, thereby inhibiting the activation of the type I interferon signaling pathway (75). This multi-target and multi-mechanism immunosuppressive effect may allow TGEV to effectively replicate in host cells and escape immune clearance, **Figure 3**.

### 3.2 TGEV escapes MDA5 recognition through cap structure modification and signal axis interference

Notably, NSPs can suppress IFN signaling activation even when segments of viral RNA are recognized, whereas modifications of the cap structure further decrease the probability of detection (76, 77). Host cells trigger the innate immune response by detecting viral RNA’s distinct features, known as “non-self” markers (78). MDA5, a key member of the RLRs family, primarily recognizes lengthy dsRNA generated during viral infections or single-stranded RNA lacking a complete 5′ cap modification (79, 80). When MDA5 recognizes PAMPs of these dsRNAs, MDA5 can directly interact with the MAVS, and then recruit and activate other downstream signaling proteins like TBK1 and IKKϵ. Finally, the IRF3/7 will be phosphorylated and their translocation to the nucleus, triggering the expression of IFN-α/β and associated ISGs. Consequently, an extensive antiviral immune response is initiated (81, 82), **Figure 3**.

To escape from the host’s innate immune attack, coronaviruses have developed mechanisms to conceal the foreign features of their RNA. One such mechanism involves NSP16, acting as an S-adenosylmethionine (SAM)-dependent 2’-O-methyltransferase. NSP16 catalyzes methylation at the 2’-hydroxyl site of the ribose on the 5’-cap structure of viral mRNA, converting the RNA cap structure from Cap- (m^7^GpppN) to Cap-1 (m^7^GpppNm) (83, 84). This modification mimics the cap structure of eukaryotic cell mRNA, thereby diminishing recognition of viral RNA by host PRRs (85). Among human coronaviruses, SARS-CoV-2 can evade host immune responses by modifiying the RNA 5′ ends of viral RNA through the modification of the non-structural protein NSP16 (86). Specifically, NSP16 forms a heterodimer with NSP10 and catalyzes 2′-O-methylation of the first ribose unit of viral mRNA, generating a Cap-1 structure. This modification allows viral RNA to mimic host mRNA, thereby preventing recognition by MDA5 and inhibiting type I interferon signaling pathways (87). Viruses such as TGEV and SARS-CoV-2 exploit NSP16-mediated 2′-O-methylation to alter their RNA in both spatial conformation and chemical properties, effectively “disguising” it as endogenous molecules and escaping MDA5-mediated detection and immune activation (88, 89). Moreover, NSP16 activity is regulated by NSP10, with the NSP16–NSP10 complex also capable of suppressing host protein translation (88, 90), **Figure 3**.

## 4 Disruption of intestinal barrier function by TGEV

### 4.1 TGEV damages the tight junctions of intestinal epithelium

The intestinal epithelial barrier is essential for preserving intestinal homeostasis and preventing the trans-epithelial infiltration of pathogens and toxins (91, 92). Tight junctions (TJs) are pivotal for maintaining the integrity of this barrier, primarily comprised of various cytoplasmic proteins and transmembrane such as Zonula occludens-1 (ZO-1), Occludin, and Claudin family proteins (93, 94). These proteins collaborate to form a sealing belt structure between neighboring cells, restricting the passage of luminal contents to the basolateral side and thereby upholding the selective permeability of the barrier (95).

Prior research has demonstrated that TGEV infection disrupts the epithelial tight junction structure significantly (96). Upon infecting the IPEC-J2 porcine small intestinal epithelial cell line, TGEV notably reduces the expression levels of tight junction-related proteins, including ZO-1, Occludin, and Claudin-1. Additionally, the localization and structural integrity of tight junction proteins are compromised, leading to the disruption of the belt-like junction and widening of the intercellular space (97, 98). These alterations notably compromise the barrier function between epithelial cells (95).

Mechanistic investigations have elucidated that TGEV triggers the upregulation of inflammatory cytokines, including TNF−α, IL−6, and IL−8, through the activation of the p38 MAPK and NF-κB signaling pathways, as evidenced by studies (99, 100). These inflammatory factors indirectly impede the transcriptional activity of tight junction proteins (96). In addition, TGEV infection was accompanied by mitochondrial dysfunction and increased oxidative stress, which were characterized by increased contents of ROS and mitochondrial membrane potential (101–103). Excessive ROS can promote the degradation of TJs proteins or abnormal localization on the cell membrane, thereby exacerbating the barrier disruption (104, 105).

**Figure 4:**
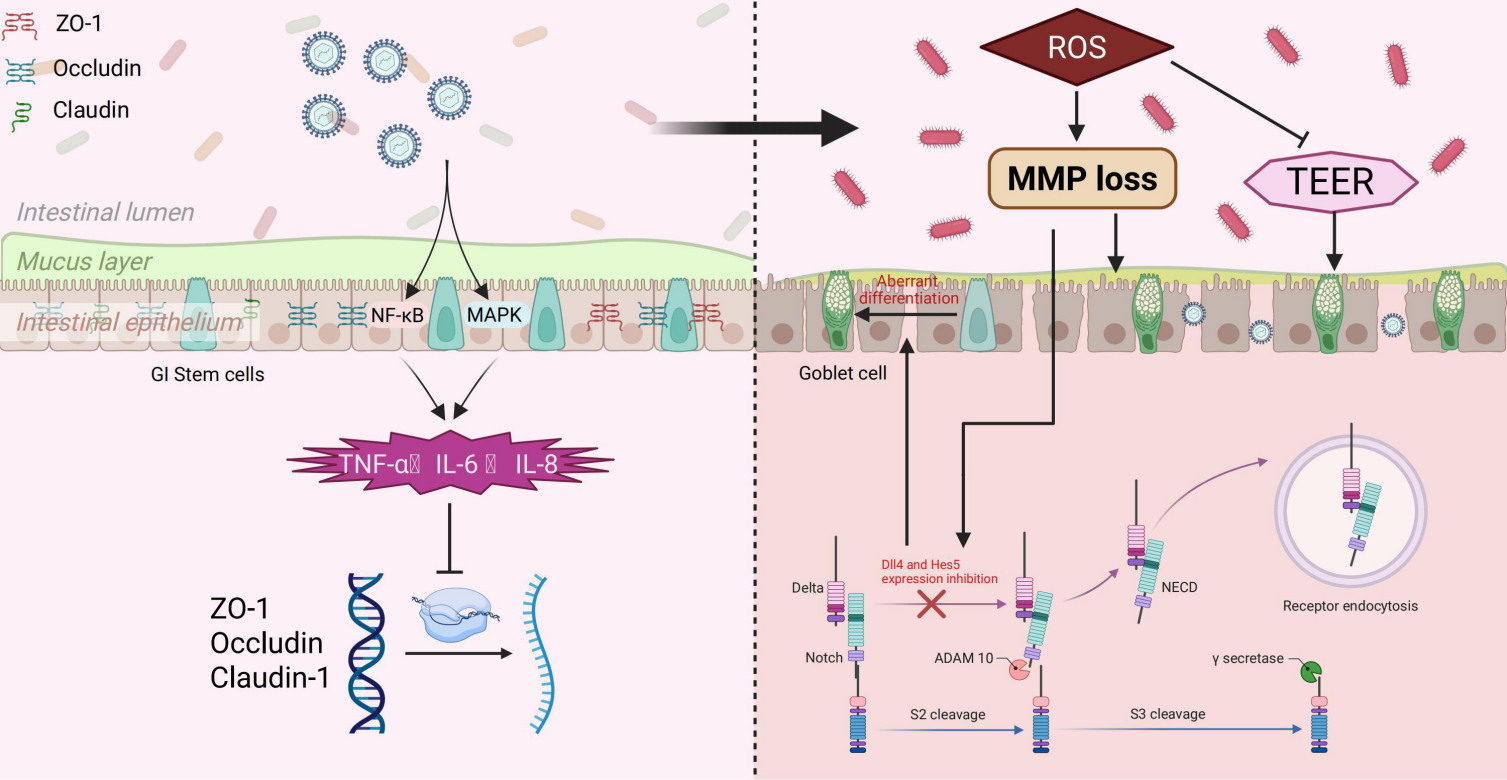
TGEV-induced disruption of intestinal barrier and modulation of Notch signaling. TGEV infection activates NF-κB and MAPK pathways, promoting inflammatory cytokine release (TNF-α, IL-6, IL-8) and downregulating tight junction proteins (ZO-1, Occludin, Claudin-1), leading to barrier dysfunction. Excess ROS impairs mitochondrial function, reduces TEER, and inhibits Dll4 and Hes5 expression in the Notch pathway, causing aberrant differentiation of GI stem cells into goblet cells, which may facilitate viral replication and spread.

Functional studies have demonstrated that TGEV infection reduces transepithelial electrical resistance (TEER) and increases the permeability of the epithelial barrier, as evidenced by enhanced leakage of fluorescent tracer molecules (96, 100). This heightened barrier permeability facilitates the translocation of luminal pathogens, such as bacteria or endotoxins, across the epithelium, leading to potential secondary infections or systemic inflammatory responses in the host (106). Furthermore, the compromised barrier function facilitates local dissemination and amplification of TGEV within the intestinal tract, thereby exacerbating disease progression and tissue damage (21), **Figure 4**.

### 4.2 TGEV regulates the notch signaling pathway

During TGEV infection of small intestinal epithelial cells, elevated levels of ROS and a loss of mitochondrial membrane potential were observed, resulting in oxidative stress (107). The stress condition could then suppress the expression of several important factors involved in the Notch signaling pathway, including Dll4 and Hes5 (108, 109). This mechanism has been demonstrated in the Paneth cell model of TGEV targeting small intestinal crypts (90, 110). The Notch signal is essential for regulating the balance between self-renewal and differentiation of intestinal Lgr5^+^ stem cells. Its inhibition can drive excessive differentiation of stem cells into goblet cells, resulting in elevated intestinal mucus secretion (90). This shift in differentiation and mucus composition may promote TGEV replication and dissemination in the small intestine. Consequently, inhibiting the Notch signal not only fails to restrict goblet cell production but also creates a more favorable environment for virus infection and transmission (90), **Figure 4**. Notch signaling is generally upregulated in hosts infected with viruses such as SARS-CoV-2, COVID-19, and PDCoV (111–113). However, it remains unclear whether the inhibition of Notch signaling observed during TGEV infection represents a unique feature of TGEV or a common characteristic of porcine coronaviruses. Further studies are warranted to elucidate the specificity and underlying mechanisms of this Notch signaling regulation.

### 4.3 Potential role of the gut microbiota in TGEV immune evasion

The gut microbiota constitutes the largest and most diverse microbial community within the host, and its metabolites, signaling molecules, and inter-microbial interactions play pivotal roles in maintaining immune homeostasis, preserving mucosal barrier function, and regulating inflammatory responses (114). Recent studies have revealed that the gut microbiota not only participates in nutrient metabolism and mucosal integrity but also modulates host susceptibility to viral infections and viral replication efficiency by regulating innate immune pathways, such as interferon (IFN) signaling. During TGEV infection, short-chain fatty acids (SCFAs) produced by the gut microbiota, especially butyrate, have been demonstrated to influence viral infection levels (115, 116). In the later stages of infection, butyrate can substantially enhance TGEV infection in porcine small intestinal epithelial cells and porcine testicular cells; however, this effect is not mediated through an increase in viral attachment or entry, but rather via interference with host antiviral immune responses.

Notably, TGEV infection is linked to a reduction in Lactobacillus gene copy numbers and an increase in Enterobacteriaceae gene copy numbers in porcine intestinal mucosal samples (117).In contrast, fecal samples show elevated relative abundances of *Lactobacillus* and *Limosilactobacillus (* 118). These discrepancies imply that TGEV infection may trigger varying degrees of dysbiosis in the local intestinal environment compared to the overall gut ecosystem.Furthermore, TGEV can lead to villous atrophy and compromise intestinal immune function, inducing epithelial-to-mesenchymal transition (EMT) and converting epithelial cells into motile, invasion-prone mesenchymal cells (119). Prolonged infection may further enhance the invasive capacity of fecal Enterococcus species toward intestinal cells, thereby altering gut microbial composition and creating conditions favorable for secondary pathogen infections. Collectively, these findings indicate that TGEV facilitates immune evasion and increases host susceptibility by disrupting the intestinal barrier and remodeling the gut microbiota.

## 5 Cell autophagy induced by TGEV

Autophagy is a highly conserved cellular degradation process dependent on lysosomes, serving to eliminate damaged organelles, misfolded proteins, and pathogens, thereby upholding cellular homeostasis, regulating energy metabolism, and responding to cellular stress, immune modulation, and disease processes (120). In the context of viral infections, autophagy is commonly seen as a defense mechanism enabling host cells to combat foreign intruders. By forming autophagosomes that merge with lysosomes, autophagy can encapsulate and degrade viral particles, thereby restricting viral replication and dissemination (121, 122). However, recent research has demonstrated that TGEV can trigger autophagy in host cells, leading to a significant increase in autophagosome formation. Paradoxically, the autophagy induced by TGEV fails to effectively eliminate the virus. Instead, it creates a favorable membrane structure that supports viral replication and assembly, ultimately facilitating viral propagation (123, 124). This observation indicates that TGEV can exploit the host autophagy machinery to facilitate crucial stages in its life cycle, underscoring a nuanced and intricate interplay between the virus and the host autophagy system (124).

Research has shown that TGEV infection triggers robust activation of the autophagic process in host cells. Following infection, there is a notable increase in the number of autophagosomes within cells, exhibiting characteristic double-membrane structures observable via electron microscopy (123, 125, 126). Additionally, Meanwhile, there is a marked increase in the conversion of the autophagy marker microtubule - associated protein 1 light chain 3 (LC3) from its cytosolic form, LC3 - I, to the membrane - bound form, LC3 - II, resulting in a significantly elevated LC3 - II/LC3 - I ratio. This phenomenon is partly attributed to the common strategy of coronaviruses hijacking LC3-associated membranes to generate double-membrane vesicles and other replication organelles, which serve as scaffolds for the assembly of viral replication–transcription complexes (RTCs). However, this process predominantly relies on LC3 - I - positive endoplasmic reticulum - derived degradation - enhancing alpha - mannosidase - like protein 1 (EDEM1) - containing membranes (EDEMosome - like membranes) rather than classical lipidated LC3 - II membranes, further supporting the notion of enhanced autophagic activity (123, 127). At the molecular level, key autophagy-related genes such as Beclin-1 are significantly upregulated following infection (127). This may reflect a host protective mechanism in mammals, where the suppression of viral autophagic protein Beclin-1 titers contributes to defense (128, 129). Targeting Beclin-1 can block autophagosome formation and vesicle nucleation, thereby promoting viral replication and disease progression.

However, the above-mentioned autophagic response does not necessarily mean that the TGEV is cleared by host cells. On the contrary, evidence suggests that the virus may exploit this process to facilitate its replication and amplification, as shown by [Zhu et al.,2016] (127, 130). Recent studies have identified transmembrane protein 41B (TMEM41B) as a pivotal regulator during TGEV infection. TMEM41B is a protein with multiple transmembrane domains that is situated in the endoplasmic reticulum, is recognized as a key player in regulating membrane lipid translocation and curvature alterations, crucial for autophagosome formation. TGEV infection leverages TMEM41B-mediated membrane remodeling to generate double-membrane vesicles (DMVs), serving as a spatial platform for viral replication and transcription. Experimental data demonstrate that depletion or inhibition of TMEM41B significantly disrupts DMV formation, consequently impeding effective TGEV replication in host cells.Furthermore, several NSPs encoded by TGEV, such as NSP3, NSP4 and NSP6, could also interact with the endoplasmic reticulum membrane system to restructure the host cell membrane architecture. These NSPs collaborate to recruit lipids, induce local membrane expansion, and facilitate endoplasmic reticulum membrane bending, promoting DMV formation to support the assembly of the replication-transcription complex (RTC) (126). Consequently, TGEV orchestrates an intracellular replication niche conducive to its life cycle by finely regulating factors associated with the host autophagy pathway and membrane dynamics (**Table 1**).

**Table 1:** Autophagy-related molecules and their functions during TGEV infection.

Molecule/Protein	Type	Post-infection changes	Main functions	The role in TGEV infection
LC3(LC3-I / LC3-II)	Autophagy marker molecules	The conversion level from LC3-I to LC3-II is elevated.	Core molecules in the formation and elongation of autophagosomes	Enhanced autophagic activity promotes the accumulation of autophagosomes, providing a platform for TGEV replication.
Beclin-1	Core components of the autophagy initiation complex	Expressing an upward adjustment	Regulation of autophagosome initiation and nucleation	The upregulation is conducive to the formation of autophagosomes and may be hijacked by TGEV to promote replication.
ATG5	Autophagy-related genes/proteins	Expressing an upward adjustment	Participate in the extension and closure of autophagosome membranes	The enhanced autophagic activity after the increase is conducive to the establishment of the viral membrane structure platform.
SQSTM1/p62	Autophagic substrates and selective receptors	Accumulation after infection	Conjugate ubiquitinated proteins and mediate their degradation through LC3	Accumulation in the mitochondrial fraction during the separation of components suggests the occurrence of mitophagy, which is specific to mitochondria.
TMEM41B	Endoplasmic reticulum multi-pass transmembrane proteins	Essential factor	Participate in membrane lipid flipping, membrane curvature changes, and promote the formation of autophagosomes.	Regulating the formation of double-membrane vesicles (DMVs) and the absence inhibits the replication of TGEV.
NSP3/NSP4/NSP6	Non-structural protein of TGEV	Expressed in infected cells	Interact with the endoplasmic reticulum membrane and restructure the membrane structure.	Coordinately induce endoplasmic reticulum membrane remodeling, promote the formation of the double-membrane vesicle (DMV), and provide a platform for the replication-transcription complex (RTC).

This table summarizes the alterations and functional roles of key autophagy markers, core autophagy-related genes, selective autophagy receptors, and viral non-structural proteins in host cells *after* TGEV infection.It highlights their involvement in autophagosome formation, double-membrane vesicle (DMV) biogenesis, and the assembly of the replication–transcription complex (RTC).

## 6 Apoptosis induced by TGEV

Apoptosis is a type of programmed cell death that is precisely controlled by genes and is broadly involved in many physiological processes such as individual development, maintenance of tissue homeostasis, and elimination of abnormal cells (131). This process is energy-dependent and exhibits highly ordered morphological and molecular biological characteristics (131). Apoptosis primarily operates through two established signaling pathways: the intrinsic pathway mediated by mitochondria and the extrinsic pathway mediated by death receptors (132, 133).

Studies have shown that infection with TGEV can trigger a robust oxidative stress response and activate cell apoptosis. During the peak of viral replication, host cells must generate significant energy and biosynthetic resources to support viral proliferation, leading to mitochondrial dysfunction and a notable increase in intracellular ROS and mtROS accumulation (103, 127, 134). Intestinal epithelial cells, being the primary targets of TGEV, exhibit a strong ability to regulate oxidative stress. However, TGEV infection results in severe mitochondrial damage in these cells, accompanied by pronounced autophagy and mitophagy activation. The ROS buildup induced by TGEV not only directly harms cell structures but also induces programmed cell death by activating the mitochondrial apoptosis pathway. Specifically, ROS-mediated stress signals trigger p53 phosphorylation, resulting in the movement of the pro-apoptotic protein Bax to the outer membrane of mitochondria, increasing mitochondrial membrane permeability, releasing cytochrome c into the cytoplasm, and subsequently activating Caspase-9 and downstream effector molecule Caspase-3, ultimately initiating cell apoptosis (103, 135). Notably, TGEV infection upregulates the expression of various antioxidant-related genes, likely as a host response to virus-induced oxidative damage (127, 135). However, approximately 12 hours post-infection, during active virus replication, substantial mitochondrial degradation and increased autophagosomes are observed, indicating persistent mitophagy activation. While mitophagy can mitigate oxidative damage to some extent, excessive activation may exacerbate cell apoptosis by depleting mitochondrial function (136). At the S and G2/M phases of the host cell cycle, TGEV’s N protein facilitates the activation of p53 and subsequently upregulates its downstream effector p21, further promoting cell death through the intrinsic apoptosis pathway (136). These processes eventually cause apoptosis of porcine small intestinal epithelial cells, leading to villi atrophy, thinning and loss of intestinal wall elasticity, gastrointestinal bleeding, barrier function damage, and finally severe diarrhea, which is a lethal symptom of TGEV infection (134).

## 7 Pyroptosis induced by TGEV

Pyroptosis is a programmed cell death mechanism that hinges on the activation of caspase-1-like proteases and is distinguished by pronounced inflammatory characteristics (137). This process is primarily triggered by the cleavage and activation of Gasdermin D (GSDMD), which subsequently facilitates the formation of membrane pores, the release of inflammatory mediators, and ultimately culminates in cell lysis and demise (138). Research has demonstrated that infection of small intestinal crypt cells, particularly Paneth cells, by the TGEV can prompt caspase-1 activation, GSDMD cleavage, and the initiation of a classical pyroptotic cascade (139, 140).

Following infection of intestinal epithelial cells by TGEV, the virus’s PAMPs, such as viral RNA, are recognized by inflammasome sensors like NLRP3 within host cells (139, 141). This recognition initiates inflammasome assembly and activates caspase-1, which subsequently cleaves GSDMD to release its N-terminal fragment (GSDMD-N). GSDMD-N then integrates into the cell membrane to form pores, disrupting membrane integrity and causing the release of cellular contents, including the inflammatory cytokines IL-1β and IL-18 (139). This cascade not only elicits a local inflammatory response in the intestine but also represents a pivotal mechanism through which TGEV induces intestinal damage and pathological alterations (141).

## 8 Summary and outlook

In recent decades, the evolutionary pace of coronaviruses has notably quickened, leading to the emergence of highly pathogenic strains like SARS-CoV, MERS-CoV, and SARS-CoV-2, which have posed significant challenges to global public health (22). The recurrent epidemics and outbreaks of novel coronaviruses have reignited interest in studying the impact of TGEV, a prototypical porcine α-coronavirus, on the swine industry and its possible zoonotic characteristics (21, 142).

TGEV employs multilayered mechanisms to interfere with host innate immune responses, thereby achieving effective immune evasion. These strategies include suppression of PRRs-mediated signaling pathways such as TLRs and RLRs to inhibit interferon production; modification of viral RNA with a cap structure to escape host RNA sensing; modulation of host signaling pathways such as Notch to dampen immune responses; disruption of the intestinal epithelial barrier and reshaping of gut microbiota to compromise barrier function; and induction of multiple forms of programmed cell death, including pyroptosis, apoptosis, and autophagy, to weaken antiviral defenses. Moreover, the TGEV genome exhibits high variability, conferring robust immune evasion capacity and limiting the cross-protective efficacy of existing vaccines, which contributes to unstable protection. Although the clinical detection rate of TGEV has decreased in recent years, this does not mean that its disappearance from natural reservoirs (143). On the contrary, owing to its highly mutable genome, substantial immune evasion ability, and limited cross-protection, TGEV continues to evolve (21). One of the key unresolved scientific questions is the precise identification of the molecular targets of TGEV nonstructural proteins (NSPs). Given that NSPs play pivotal roles in suppressing host innate immunity, yet their specific targets and mechanisms remain incompletely understood, elucidating these interactions will offer an important theoretical foundation and potential intervention strategies for mitigating viral immune evasion. In addition, a complex interplay exists between enteric viruses and the host gut microbiota, which may critically modulate viral immune evasion. Therefore, strategies designed to minimize or prevent the disruption of gut microbial homeostasis induced by TGEV represent a crucial direction for future therapeutic development.Elucidating the tripartite interactions among TGEV, the microbiota, and the host will not only enhance our understanding of TGEV pathogenesis but also provide theoretical foundations for the development of novel microbiota-based interventions against TGEV. Notably, TGEV has traditionally been considered strictly host-specific, confined to infecting swine species. However, increasing evidence indicates that coronavirus host barriers are not absolute, and their cross-species transmission potential may have been substantially underestimated (144). Recent studies have unveiled the molecular basis of TGEV cross-species transmission, showing that the receptor-binding domain (RBD) of TGEV can interact with aminopeptidase N (APN) from 17 different species, with eight demonstrating relatively high binding efficiency. This finding underscores the potential of TGEV for cross - species transmission, raising concerns regarding the possibility of human infection.

Currently available TGEV vaccines, which predominantly rely on traditional platforms, are confronted with multiple limitations, encompass insufficient protective efficacy, restricted cross-protection, risks of reversion to virulence, and immune interference. Consequently, there is an urgent demand for more advanced and effective preventive strategies. Future research directions may include: (i) rational design of broad-spectrum coronavirus vaccines based on conserved antigenic epitopes of TGEV; (ii) exploration of novel mucosal adjuvants and efficient delivery systems, coupled with genetic engineering or pharmacological induction to enhance host antiviral effectors (e.g., upregulating of GSDMD) to improve intracellular pathogen clearance; and (iii) rational attenuation strategies and targeted drug development to inhibit key viral immune evasion proteins (e.g., NSP1, ORF6), thereby blocking their interference with host immune signaling.Furthermore, given the potential cross-species transmission risk of TGEV and other coronaviruses, the establishment of comprehensive surveillance and prevention systems is essential. Such systems should encompass continuous monitoring and tracing of viral recombination events, scientific evaluation of human susceptibility, and integration of human, animal, and environmental health management under a “One Health” framework. This integration will enable proactive prevention and rapid response to potential public health threats.

Future research should therefore prioritize core scientific issues, including: (i) the immune evasion targets of nonstructural proteins (e.g., NSP1, NSP3, ORF6); (ii) the impact of virus–microbiota interactions on immune evasion; (iii) the cross-species transmission potential of TGEV and its implications for human health; and (iv) the risk of generating novel viral populations through recombination with other porcine coronaviruses. Addressing these questions will provide critical guidance for vaccine development, intervention strategies, and public health preparedness. The advancement of state-of-the-art technologies, such as single-cell sequencing, spatial transcriptomics, proteomics, and CRISPR screening, is enhancing the precision of constructing TGEV infection models and target screening. This progress is anticipated to advance translational research on antiviral drugs and intervention strategies (145). Future optimization efforts may involve integrating innovative adjuvant technologies, nanodelivery systems, mucosal immunity strategies, and novel vaccines utilizing virus-like particles (VLPs) and mRNA platforms. Additionally, vigilance in monitoring recombination and evolution events between TGEV and other porcine coronaviruses (e.g., PEDV, PDCoV, SADS-CoV) is crucial for early detection of emerging coronaviruses (146). Despite the reduced clinical impact of TGEV amid frequent zoonotic diseases, its potential for ongoing recombination and evolution poses indirect threats to human health that cannot be disregarded (146, 147). Consequently, comprehensive research on the mechanisms of cross-species transmission, evolutionary dynamics, and potential public health risks associated with porcine coronaviruses should be intensified to enable early detection and effective management of potential emerging zoonotic viruses.
